# Complete and Rapid NMR Characterization of Therapeutic Monoclonal Antibodies via Differential Labeling of Fab Fragments in *Escherichia coli*

**DOI:** 10.1021/acs.analchem.6c00109

**Published:** 2026-04-07

**Authors:** Donald Gagné, Yves Aubin

**Affiliations:** † Centre for Oncology, Radiopharmaceuticals and Research, Biologics and Radiotherapeutic Drugs Directorate, Health Canada, 251 Sir Frederick Banting Driveway, Ottawa, ON K1A 0K9, Canada; ‡ Department of Chemistry, Carleton University, 1125 Colonel By Drive, Ottawa, ON K1S 5B6, Canada

## Abstract

Characterization of therapeutic monoclonal antibodies (mAbs) structure and dynamics using NMR spectroscopy has benefited from recent advances in sample preparation methods for isotopically labeled Fab and Fc fragments. Here, we propose a simple and accessible method using *E. coli* to produce the heavy and light chains separately, thereby allowing differential labeling of either chain of the refolded fragment. This was demonstrated on the Fab fragments of adalimumab, bevacizumab, infliximab, rituximab, and trastuzumab. The new labeling scheme produced NMR spectra with almost no resonance overlap, allowing near-complete assignments (>90%) of backbone resonances in less than an hour. In addition, a near-complete assignment of side-chain methyl groups was carried out that allowed the assignments of Avastin. The total time from Petri dish to final backbone assignment was 5 weeks per chain.

## Graphical abstract

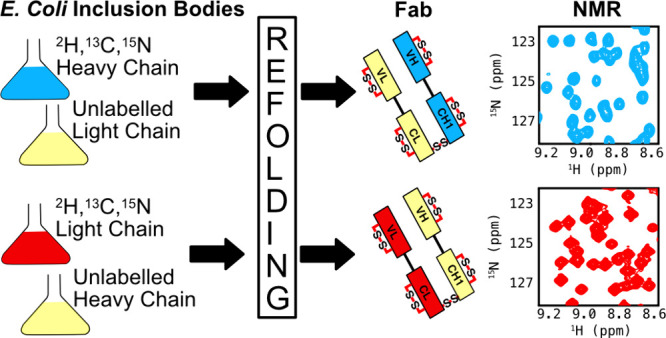

It is well recognized that therapeutic monoclonal antibodies (mAbs) make up an important and fast-growing class of biological drugs. The complexity of their molecular structure requires an array of physicochemical techniques and biological assays to evaluate the biologically active conformation, often referred to as the higher-order structure (HOS). While descriptions of mAbs at atomic details can be provided by X-ray crystallography, it is neither practical nor feasible in this context. Nuclear magnetic resonance spectroscopy (NMR) can provide high-resolution assessments of the HOS of small therapeutic proteins and mAbs using techniques at natural abundance.^1–4^ The comparison of 2D NMR spectra between a candidate protein drug and a well-characterized reference (innovator) protein, such as in the assessment of biosimilars or when significant manufacturing changes of innovator products are required, provides invaluable information on the comparability of their molecular structure (primary, secondary, and tertiary). When applied to small proteins where all resonances can be assigned, such as cytokines and hormones, this exercise provides an assessment at the atomic level of the molecular structure. However, when a resonance assignment of the NMR spectra is not available, such as therapeutic mAbs, assessment of the structure must rely on statistical analysis of the complex pattern afforded by the 1D and 2D NMR spectral maps. Obtaining complete resonance assignments for mAbs has long been impeded by the challenge to incorporate carbon-13 and nitrogen-15, but more importantly, deuterium isotopes. The replacement of carbon-bound protons by deuterons is essential to allow the collection of 3D NMR data sets on these large 50 kDa Fab and Fc fragments. For many years, attempts at backbone resonance assignments have been limited to the crystallizable fragment (Fc) of mAbs.^5,6^ Recently, approaches for the production of isotopically labeled antigen-binding fragment (Fab) of mAbs using yeast, bacteria, and cell-free systems have been reported by us and other groups.^7–9^ In all cases, near-complete resonance assignments of the Fab have been obtained^10–12^ using either of these labeling approaches.

Antibody fragments, whether they are Fab or Fc, are somewhat complex, yet very stable protein scaffolds made of two polypeptide chains with two immunoglobulin domains each per chain that are stabilized with a disulfide bridge. In addition, both chains are connected by a disulfide linkage. For these reasons, expression systems based on eukaryotic cells are used for the production of soluble mAbs and their fragments to ensure proper folding and the correct topology of the disulfide linkages. However, isotopic enrichment is very challenging in these systems. Apart from the yeast *Komagataella phaffii*, incorporation of deuterium on carbon-bound proton to reduce transverse relaxation times to allow NMR data collection for resonance assignments or spin relaxation studies is not feasible in eukaryotic systems. In contrast, expression of heavy and light chains of Fab fragments in the form of inclusion bodies in *E. coli* is straightforward, and has been achieved at high yields.^8^ In our experience, refolding mAb fragments produced as inclusion bodies in *Escherichia coli* is the method of choice.

Here, we report a simple, economical, and accessible method for the selective labeling of either heavy or light chain of Fab fragments that greatly accelerates the NMR resonance assignments procedure from months to hours. The approach is based on the separate production of the heavy and the light chains in *E. coli* that, after isolation, are mixed together prior to refolding. This offers the advantage of performing differential chain labeling where only one chain (either the heavy or the light chain) per fragment is isotopically labeled, hence, reducing by half the number of amide resonances, which nearly eliminates peak overlap, resulting in a straightforward and rapid assignment task.

Initially, the approach, which consists of mixing two polypeptide chains, was tested on the Fc fragment of trastuzumab. All IgG1 therapeutic mAbs share the exact same primary sequence on their Fc, except at residues 356 and 358 (using the numbering of bevacizumab), where some, such as bevacizumab and trastuzumab, have a glutamate and methionine, respectively, while others such as adalimumab, infliximab, and rituximab, have an aspartate and a leucine, respectively. After successful production of this Fc and NMR resonance assignment, the Fab fragments of adalimumab, bevacizumab, infliximab, rituximab, and trastuzumab were produced using this approach.

## Experimental Section

Sample production is described briefly below, with very detailed procedures provided in supporting infomormation. DNA constructs coding for the heavy chains of adalimumab-Fab, bevacizumab-Fab, infliximab-Fab, rituximab-Fab, trastuzumab-Fab, infliximab-Fc, and bevacizumab-Fc and their corresponding light chains were synthesized commercially with optimization for expression in *E. coli*, and inserted using the *Nde*I and *Bam*H1 restriction sites in the pET11a vector by the manufacturer (BioBasic). Expression of each chain and isotopic labeling was carried out exactly as described previously.^8^ The cell pellets, one for each chain (light and heavy), were reconstituted with 35 mL of lysis buffer (20 mM Tris-HCl, 2 mM EDTA, 10 mM DTT, pH 8.5), and sonicated as described previously.^8^ Inclusion bodies were recovered by centrifugation at 30,000*g* for 30 min (4 °C). Each pellet was resuspended by agitation in 20 mL of buffer (100 mM Tris-HCl; 6 M GdmCl; 2 mM EDTA; 80 mM reduced glutathione; 2.5 mM DTT, pH 8.5) for 2–3 h at 4 °C. The pellet mix (heavy:light 1:1.25 or 1:2) was added dropwise at rate of 30 mL/h using a syringe pump, to the refolding buffer (20 mM Tris-HCl; 1 M l-arginine-HCl; 3.2 mM oxidized glutathione; pH 10.0). Protein density was kept at 100 μg/L or less to minimize unwanted intermolecular interaction in order to maximize the refolding efficiency with an oxidized-to-reduced glutathione ratio of ≥4. Refolding solution was stirred gently at 4 °C for 65–70 h. Prior to purification of the refolded fragments, extensive centrifugations of the solution followed by filtrations were carried out to remove any protein precipitates resulting from impurities and misfolded species. Once a clear solution exempt of any turbidity was obtained, the fragments were purified using affinity chromatography and cation exchange chromatography as described previously.^8^ For the four Fab and the two Fc tested, *E. coli* cultures of 0.5–1.0 L for each chain (heavy and light) yielded, on average, 40–60 mg of properly folded isotopically labeled Fab ready for NMR data collection. For proteins with molecular weights of 30 kDa and higher, it is current practice to use uniformly labeled ^2^H-^13^C-glucose and deuterium oxide as solvent for the growth media to replace all carbon-bound protons with deuterons to the highest level possible (>97%) to attenuate transverse relaxation of carbon resonances during the execution of 3D NMR pulse sequences to maximize signal intensities. In parallel, we also used the less expensive ^13^C-glucose as a carbon source that leads to a lower level of deuteration of about 80–85% when media is prepared with deuterium oxide (D_2_O) as solvent.^13^ In addition, the side-chain methyl groups of valines, leucines, and isoleucines-δ1 were labeled using the standard protocol.^14^ All samples contained 350 μL of 450–500 μM fragments in 5 mm Shigemi tubes.

A sample of Avastin for NMR was prepared^2^ by passing the formulated product through a Protein A column and eluted with 0.1 M glycine-HCl buffer pH 3.0. Fractions containing the antibody were pooled, buffer exchanged with 20 mM sodium acetate-d_3_ pH 5.0, and concentrated to 550 μL. Deglycosylation (hydrolysis of the glycosidic bond between the two *N*-acetyl-glucosamine moieties) of Avastin was carried out using Endo-S2 produced in-house according to Li et al.^15^ and Hatfield et al.^16^

All data collection was carried out at 40 °C on Bruker NEO 600 MHz (side-chain methyls), AVANCE III-HD 700 MHz (backbone assignments), and AVANCE II 900 MHz (some initial 2D-HSQC) instruments, all equipped with 5 mm TCI cryoprobes. Data collection for the assignment of backbone resonances was carried out using the TROSY version with deuterium decoupling of the standard pulse sequences from the Bruker library (Table 1).

**Table 1:** 3D NMR Experiments for Backbone Resonance Assignments

Experiment	Pulse sequence	Spectral windows (^1^H/^15^N/^13^C) in ppm
3D-Tr-HNCO	trhncogp2h3d	18/40/14
3D-Tr-HN(CA)CO	trhncacogp2h3d	18/40/14
3D-Tr-HNCA	trhncagp2h3d2	18/40/30
3D-Tr-HN(CO)CA	trhncocagp2h3d)	18/40/30
3D-Tr-HNCACB	trhncacbgp2h3d	18/40/80
3D-Tr-HN(CO)CACB	trhncocacbgp2h3d	18/40/80

All 3D data matrices were collected with 2048 total points in the direct dimension (^1^H), 64 total points in the nitrogen dimensions and 128 total points in the carbon dimensions. Using a relaxation delay of 1.2 s, 16 transients per FID, and the parameters of Table 2, the total acquisition time was 52 h per 3D data set. Nonuniform sampling schemes were not utilized to maximize sensitivity. Data collection for the assignment of the methyl resonances was carried out using four 3D experiments 3D-CCC­(CO)­NH, 3D-HCC­(CO)­NH, 3D-CCC­(CA)­NH, and 3D-HCC­(CA)­NH that were graciously provided by Prof. Lewis Kay (University of Toronto).^17^ Data were collected with a spectral window of 16 ppm with 2048 real points in the proton direct dimension and a spectral window of 40 ppm with 64 real points in the nitrogen dimension centered at 120 ppm. The indirect proton dimensions in the (HCC−) experiments were collected with a spectral window of 3 ppm with 64 real points and the indirect carbon dimensions of the (CCC−) experiments with a spectral window of 22 ppm with 54 real points for a total acquisition time of 54 and 64 h, respectively. Chemical shift resonances were referenced with sodium 2,2-dimethyl-2-silapentane-5-sulfonate (DSS). NMR data were processed using nmrPipe^18^ using the NMRBox web facility^19^ and visualized with NMRViewJ.^20^ Semiautomated sequential assignment was carried out with the Runabout tool of NMRViewJ software.

**Table 2:** All Assignment Statistics

	Backbone^a^					Methyls^b^				
	CA	CB	CO	N	H	Ala	Ile	Leu	Thr	Val
Bevacizumab-Fab										
U-^13^C-glucose										
Light chain	97%	95%	93%	95%	95%	11/11	7(6)/7	14/14	16/19	14/16
Heavy chain	87%	87%	87%	85%	85%	11/15	2(0)/2	16/17	18/22	18/21
U-^2^H-^13^C-glucose										
Light chain	99%	98%	99%	98%	98%	N/A	7(0)/7	14/14	N/A	15/16
Heavy chain	88%	85%	88%	85%	85%	N/A	2(0)/2	17/17	N/A	20/21

Trastuzumab-Fab										
U-^13^C-glucose										
Heavy chain	91%	88%	91%	89%	89%	15/16	4/5	13/16	19/20	20/21

Rituximab-Fab										
Heavy chain	95%	95%	95%	95%	95%	I.P.^c^	6 (I.P.)	13/13	I.P.	14/16

Crystallizable Fragments										
FcDEL	87%	78%	86%	91%	91%	6/7	4/4	18/18	14/15	20/23
FcEEM	89%	85%	87%	90%	90%	4/7	4/4	16/17	15/15	21/23

a Percentages are for non-proline residues for amides (H, N) and non-glycine residues for CB.

b Number/total refers to the number of assigned methyl groups over the total expected. For alanines: Hβ/Total; isoleucines: Hδ1­(Hγ2)/Total; leucines: Hδ1­(Hδ2)/Total; threonines: Hγ2/Total; valines: Hγ1­(Hγ2)/Total.

c I.P. = in progress.

## Results and Discussion

High yields of purified fragments were obtained for all Fab and Fc fragments attempted (adalimumab-Fab, bevacizumab-Fab, infliximab-Fab, rituximab-Fab, and trastuzumab-Fab, infliximab-Fc, and trastuzumab-Fc) with this method (Supplemental Figures 1 and 2), while the single chain approach^8^ failed to produce bevacizumab-Fab or infliximab-Fab.

Differential labeling of bevacizumab allowed the effective separation of resonances of the heavy and light chains in NMR spectra that resulted in nearly fully resolved 2D proton–nitrogen correlation maps (Figure 1). Data collection of the standard 3D NMR experiments yields high-sensitivity and highly resolved data sets that greatly facilitated the daunting task of backbone resonance assignment^5,6,10–12^ into a simple connect-the-dots procedure, with both carbon sources (^2^H-^13^C-glucose and ^13^C-glucose). The first pass through the data during the assignment procedure, i.e., going through all amide sets, allowed the assignment of nonproline backbone amides of infliximab-Fc (356-DEL-358) and bevacizumab-Fc (356-EEM-358) to 91% and 90%, respectively (Table 2).

**Figure 1:**
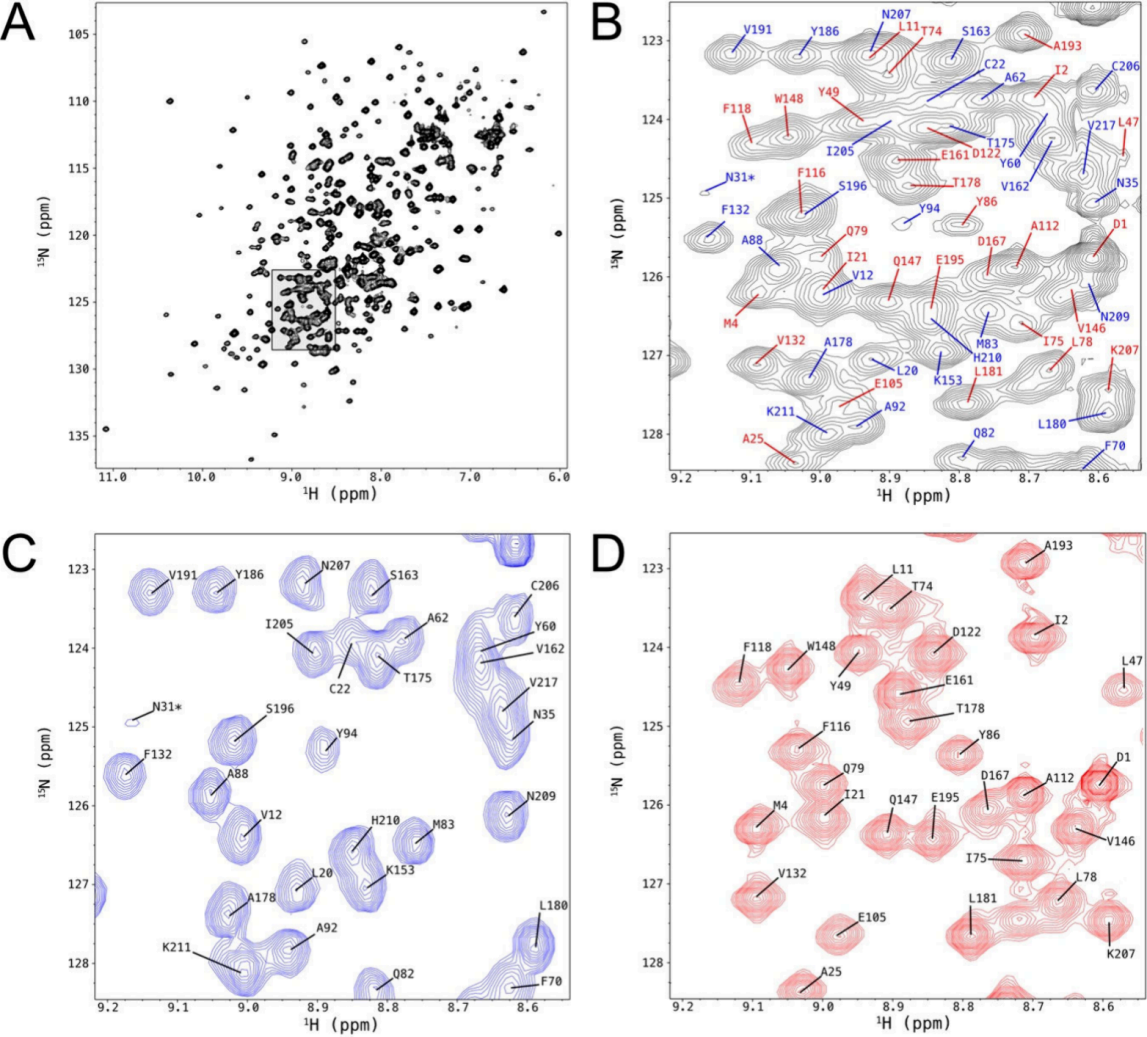
2D ^1^H–^15^N HSQC NMR spectra of ^13^C-Met-ILV-^2^H-^13^C-^15^N-bevacizumab-Fab recorded at 700 MHz in 20 mM Na-OAc-d_3_, pH 5.0 at 40 °C. In panel A, the full spectrum of bevacizumab Fab with both chains labeled. Panel B shows the expansion of the spectrum in A, indicated by the rectangle, with the assignments of the heavy chain colored blue and the light chain in red. Panels C and D show the same expansion of the assigned ^13^C-Met-ILV-^2^H-^13^C-^15^N-bevacizumab-Fab-HeavyChain and ^13^C-Met-ILV-^2^H-^13^C-^15^N-bevacizumab-Fab-LightChain, respectively. Differential labeling of the two chains allows the resolution of heavy chain V12, N207, and S196 and light chain I21, L11, and F116 that are overlapped in panel B.

Assignments have been deposited in the BioMagResBank (BMRB) with entry numbers 53199 (FcDEL) and 53197 (FcEEM). Fc fragments have a large number (20) of proline residues that induce breaks in the assignment. Using the assignment for FcEEM (BMRB 53197), we have assigned the spectra of the four glycoforms (G0, G1(6), G1(3), and G2) of this fragment that had been collected for a previous study.^16^ The assignments have been deposited in the BMRB with accession number 53625. For Fab fragments, after the first pass through all correlations to match amide residues, >85% of the nonproline backbone amides were unambiguously assigned for the heavy chain of bevacizumab-Fab, and >95% for the light chain of bevacizumab-Fab (BMRB entry number 53440). The assignment procedure (first pass) was accomplished in less than 1 h using the computer-assisted Runabout module of the NMRView software.^21^ The lower assignment rate of the heavy chain compared with the light chain of bevacizumab-Fab was attributed to the presence of slow-intermediate loop motions of the complementarity-determining regions (CDRs) (Figure 2). In order to address this lower rate, we recorded a 3D-HNCACB at 50 °C, and we prepared a sample of bevacizumab-Fab with the heavy chain triply labeled using ^2^H-^13^C-glucose and carried out *de novo* assignment. Neither of these attempts resulted in a better rate of first pass assignments. Analysis of trastuzumab-Fab labeled only on the heavy chain also showed a lower rate of assignment in the third CDR that is comparable to the initial assignment of trastuzumab-Fab and adalimumab-Fab reported earlier.^10,11^ In contrast, the assignment of rituximab-Fab (BMRB entry number 53491) was near-complete in CDR-H3 (Figure 2). Examination of the X-ray structures of AVASTIN (PDB ID 6BFT) and rituximab (PDB ID 4KAQ) shows that the CDR-H3 of the bevacizumab is unstructured, while this region adopts a two-strand β-sheet in rituximab. These observations suggest that the third heavy chain CDRs of bevacizumab-Fab and trastuzumab-Fab may experience slow-to-intermediate motions that broaden signal intensities, while rituximab has more conformational rigidity in this region.

**Figure 2:**
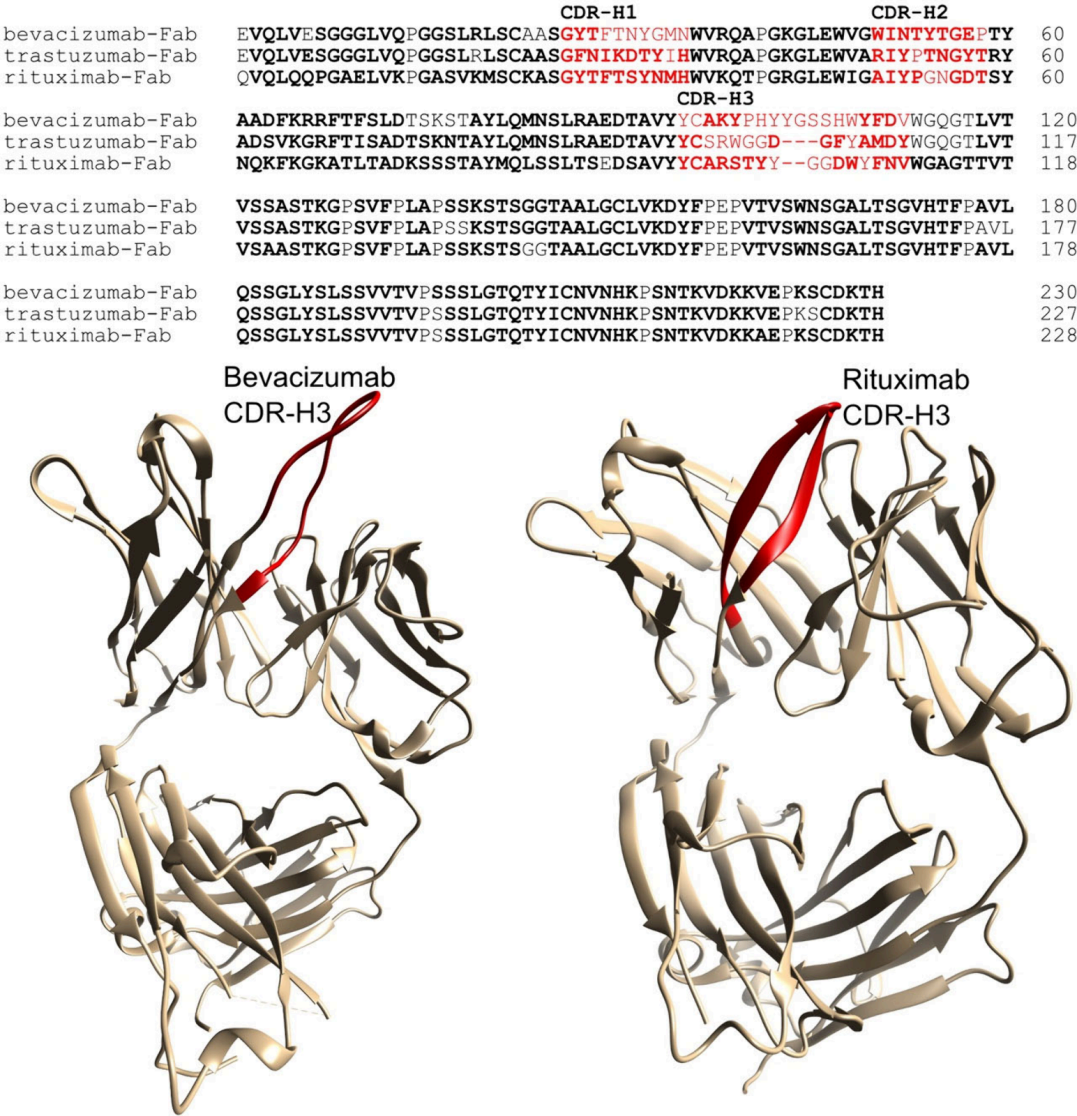
Top: Sequence alignment of bevacizumab-Fab, trastuzumab-Fab, and rituximab-Fab heavy chains showing the assigned amide residues (in bold) and the complementarity-determining regions (CDRs) H1–H3 (in red). In all therapeutic mAbs, CDR-H3 is the region with the greatest variability in amino acid types, insertions, and deletions. Bottom, CDR-H3 is shown in red on the ribbon structure of bevacizumab (left, PDB ID 6BFT) and rituximab (right, PDBID 4KAQ).

Assignment of side-chains methyl groups of valines, leucines, and isoleucines-δ1 was carried out as described previously.^10,11^ Samples prepared with ^13^C-glucose produced spectra where protons of side-chain methyl groups were incompletely exchanged with deuterons, leading to the presence of all isotopomers (^13^CH_3_, ^13^CH_2_D, and ^13^CHD_2_). These allowed the assignment of a significant number of side-chain methyl groups of alanine, threonine-γ2, and isoleucine-γ2 (Table 2) residues. In many cases, the fully protonated methyl groups (CH_3_) had a very weak signal intensity that prevented accurate measurement of their proton and carbon chemical shifts. However, isotopomers (^13^CH_2_D, ^13^CHD_2_) had sufficient signal intensities to allow their identification of the methyl signal on the 2D ^13^C­(20%) HSQC spectrum (Figure 3).

**Figure 3:**
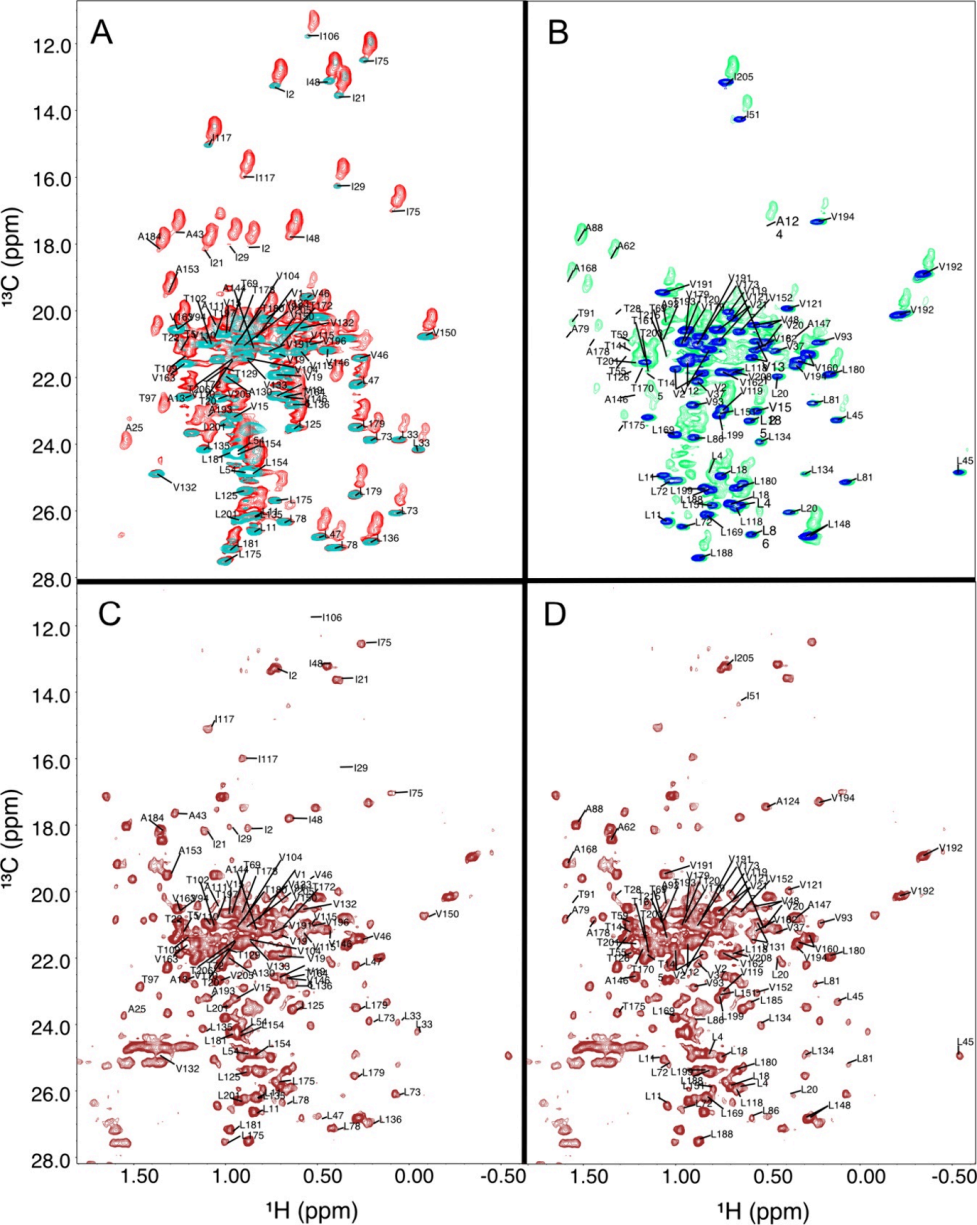
2D ^1^H-^13^C HSQC spectra of the methyl region of bevacizumab-Fab collected at 40 °C. A) In cyan, signals of ILV-^2^H-^13^C-^15^N-bevacizumab-Fab-heavy-chain (prepared with ^2^H-^13^C-glucose and labeled side-chain precursors α-keto-isobutyrate and α-keto-isovalerate, see ref 8). Here, only the side-chain methyl groups of isoleucine-δ1, leucine, and valine are protonated and can be unambiguously assigned. In red, signals of the 2D-^1^H-^13^C-HSQC of ILV-^2^H-^13^C-^15^N-bevacizumab-Fab-heavy-chain prepared with ^13^C-glucose. The slanted elongated shape of the red resonances arises from the presence of methyl isotopomers, namely CH_3_ (very weak intensity for alanine, threonine, and isoleucine-γ2), ^13^CH_2_D, and ^13^CHD_2_. In contrast, the resonances of methyl groups that originated from the labeled side-chain precursors α-keto-isobutyrate and α-keto-isovalerate are single peaks from the CH_3_ protons only. This indicates that isoleucine-δ1, leucine, and valine methyl groups do not come exclusively from these α-keto precursors but are also produced from glucose. B) Same spectral overlay for the ILV-^2^H-^13^C-^15^N-bevacizumab-Fab-light-chain. C) 2D-^1^H-^13^C-HSQC of the methyl region of ^13^C­(20%)-bevacizumab-Fab with assignments of the heavy chain. D) Same spectrum as A but with the assignment of the light chain.

From all of our results described above, we propose that NMR spectroscopy can fully characterize a therapeutic mAb by following this methodology. At first, two samples labeled with nitrogen-15 and 20% carbon-13, one for the heavy chain and the second for the light chain. These two samples provide high-resolution spectra of amides and side-chain methyl groups, from which accurate chemical shifts can be measured. Then, two triply labeled samples (^2^H,^13^C,^15^N), one labeled on the heavy chain and a second labeled on the light chain, using ^2^H-^13^C-glucose and the ILV protocol^14^), allow near-complete assignment of backbone and methyl resonance of isoleucine-δ1, leucine, and valine (Table 2). Finally, two samples (same as the second step but using ^13^C-glucose and the ILV protocol^14^) are used to complete the assignments of methyl resonances of alanine, threonine-γ2, and isoleucine-γ2 side-chains. We have shown (see Table 2) that a very high percentage of assignments for the backbone can be achieved with the use of deuterium oxide and singly labeled glucose. However, many correlations have significantly weaker intensities that lower assignment statistics, and that could impede the use of nonuniform sampling strategies for faster data collection. Considering that NMR samples of 400–500 μM for all five mAb-Fabs were produced with cultures of 0.25–0.50 L. Therefore, skipping the production and analysis of samples made with doubly labeled glucose might not be a good saving in some cases.

The direct application of this methodology is the resonance assignment of therapeutic monoclonal antibodies. The assignments of side-chain methyl groups of the Fab heavy and light chain and the Fc fragments of bevacizumab were transferred on the spectrum of the methyl region of the 2D ^1^H-^13^C HSQC of Avastin at natural abundance collected at 50 °C (Figure 4). The transfer was carried out by recording 2D ^1^H-^13^C HSQC spectra of the Fab and Fc fragments by varying the temperature with 2.5 °C increments from 40 to 50 °C and following chemical shift changes with temperature. The temperature of 50 °C was chosen because mapping the assignments on the 2D correlation map collected at 40 °C showed that many resonances have very low intensities or are simply not observed, resulting from the very slow tumbling of this 150 kDa molecule. 2D NMR spectra collected at 40 °C before and after showed no detectable changes of the methyl resonances. Heterogeneity arising from glycosylation of the drug product procured from the marketplace was addressed by removing glycans with endoglycosidase-S2 (see Figure 4C).

**Figure 4:**
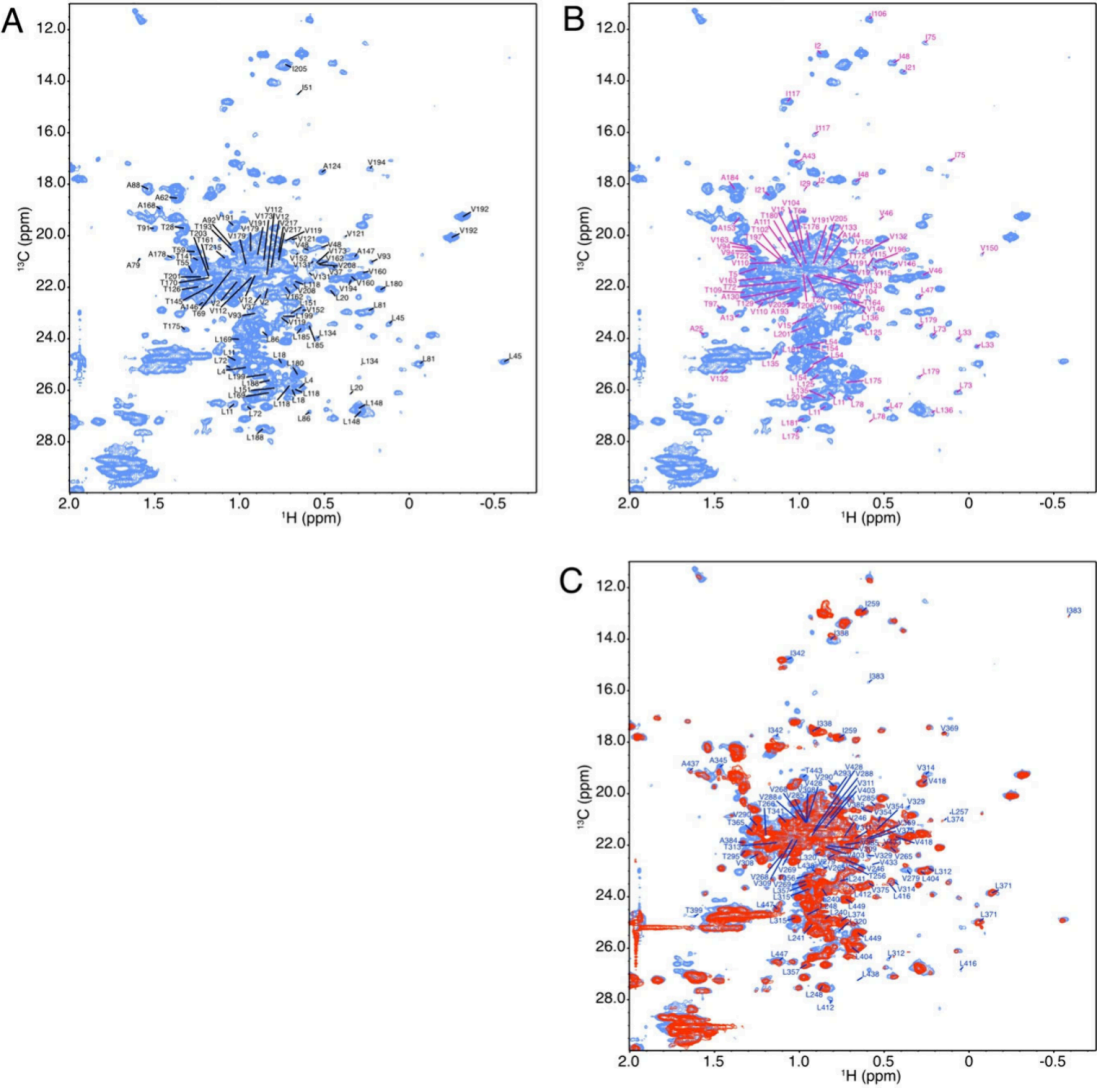
Resonance assignment of the methyl region of Avastin (bevacizumab) purchased from the local pharmacy. The NMR sample was prepared by the isolation of the active pharmaceutical ingredient (API) from the formulated product by affinity purification using a Protein-A column. After buffer exchange with 20 mM sodium acetate-d_3_, pH 5.0, and concentration, 2D ^1^H-^13^C HSQC spectra were recorded at natural abundance at 700 MHz at 50 °C. A) 2D ^1^H-^13^C HSQC spectrum of 400 μM Avastin in acetate buffer with the assignments of alanine, threonine, isoleucine, leucine, and valine methyl side-chain of the heavy chain. B) Same spectrum as A with assignments of the light chain. C) Overlay of Avastin API (blue) and deglycosylated Avastin with Endo-S2 (red) with the assignment of the methyl of the Fc fragment.

## Conclusion

The simple approach reported here removes the barriers that were impeding the study of monoclonal antibodies by NMR or any other techniques. Production of these antibody fragments is now accessible to any laboratory with basic skills in protein expression with *E. coli*. Moreover, the resulting high-resolution NMR spectra of fragments produced with this method greatly simplify and accelerate the assignment procedure while using the basic tridimensional techniques accessible to the non-NMR expert. The total time from the initial transformation of the *E. coli* cells to the backbone assignment was only 5 weeks per chain. One extra week was required to assign all side-chain methyl groups. Easy access to complete resonance assignment will enhance the usefulness of NMR of mAbs by allowing in-depth investigations such as epitope binding interactions, drug–excipient interactions, excipient-induced changes in protein dynamics, and localization of chemical modifications on mAbs sequences, to name only these.

## Supplementary Material



## Data Availability

Chemical shifts and Bruker raw data ser files and parameters were deposited in the BioMagResBank data bank with entry numbers 53440 (bevacizumab-Fab), 53491 (rituximab-Fab), 53199 (FcDEL), 53197 (FcEEM), and 53625 (glycosylated forms G0, G1(6), G1(3), and G2 of FcEEM).

## Notes

Detailed protocols for the expression, refolding, and purification of Fab and Fc fragments and 2D ^1^H–^15^N HSQC NMR spectra of adalimumab-Fab, bevacizumab-Fab, infliximab-Fab, rituximab-Fab, and trastuzumab-Fab (PDF)

## Abbreviations

mA: monoclonal antibody
scFa: single-chain fragment antigen-binding
Fa: fragment antigen-binding
Fc: fragment crystallizable
CDR: complementarity-determining regions
V_H_: heavy chain variable domain
V_L_: light chain variable domain.
C_H_1: constant domain heavy chain 1
C_H_2: constant domain heavy chain 2
C_H_3: constant domain heavy chain 3
C_L_: constant domain light chain
