# Supplementary material for: Complete and Rapid NMR Characterization of Therapeutic Monoclonal Antibodies via Differential Labeling of Fab Fragments in Escherichia coli
Source: Anal Chem. 2026 Apr 7;98(15):11322–9. doi: 10.1021/acs.analchem.6c00109 (PMC13103933; doi:10.1021/acs.analchem.6c00109)

Supporting Information

Complete and Rapid NMR Characterization of Therapeutic Monoclonal Antibodies via Differential Labelling of Fab fragments in *Escherichia coli*

Donald Gagné[1] and Yves Aubin[1,2]

1- Centre for Oncology, Radiopharmaceuticals and Research, Biologics and Radiotherapeutic Drugs Directorate, Health Canada, 251 Sir Frederick Banting Driveway, Ottawa, ON, Canada, K1A 0K9

2- Department of Chemistry, Carleton University, 1125 Colonel By Drive, Ottawa, ON, Canada, K1S 5B6

Corresponding author: Yves Aubin

Email: yves.aubin@hc-sc.gc.ca

Donald Gagné: 0000-0002-3938-6567

Yves Aubin: 0000-0002-8554-0297

I. PROTEIN EXPRESSION

A. SOLUTIONS

1. MEDIUM LURIA-BERTANI (LB)

Reagents	1000 mL
Tryptone	10 g
Yeast Extract	5 g
Sodium Chloride (NaCl)	10 g
MilliQ-water	up to 1000 mL

a) Adjust the pH to 7.0 with Sodium Hydroxide (NaOH).
b) Autoclave.

2. AGAR-LB PLATES

Reagents	1000 mL
Tryptone	10 g
Yeast Extract	5 g
Sodium Chloride (NaCl)	10 g
Agar	15 g
MilliQ-water	up to 1000 mL

a) Adjust the pH to 7.0 with Sodium Hydroxide (NaOH).
b) Autoclave.
c) At approximately 65℃, add 100 µg/mL of ampicillin.
d) Mix and pour on plates.
e) Let them jellify at room temperature.
f) Transfer to 4℃ for storage.

3. 1 M MAGNESIUM SULFATE ($MgSO_4$)

Reagents	1000 mL
Magnesium Sulfate Heptahydrate	246.5 g
MilliQ-water	up to 1000 mL

a) Autoclave.

4. 0.1 M Calcium Chloride ($CaCl2$)

Reagents	1000 mL
Calcium Chloride	14.7 g
MilliQ-water	up to 1000 mL

a) Autoclave.

5. 10X M9 Minimal Salts (Natural Abundance)

Reagents	1000 mL
Sodium Phosphate Dibasic (Na_2HPO_4)	67.8 g
Potassium Phosphate Monobasic (KH_2PO_4)	30 g
Sodium Chloride (NaCl)	5 g
MilliQ-water	up to 1000 mL

a) Adjust the pH to 7.4 with Hydrochloric Acid (HCl) or Sodium Hydroxide (NaOH).
b) Autoclave.

6. 1X M9 Minimal Medium (Natural Abundance)

Reagent	100 mL	1000 mL
milliQ-water	90 mL	900 mL
10X M9 Minimal Salts (Natural Abundance)	10 mL	100 mL
0.1 M Calcium Chloride ($CaCl_2$)	0.1 mL	1.0 mL
1 M Magnesium Sulfate ($MgSO_4$)	0.1 mL	1.0 mL
MEM Vitamins Solution	1.0 mL	10.0 mL
Ammonium Chloride (NH_4Cl) or Ammonium Sulfate $(NH_4)_2SO_4$	100 mg	1.0 g
Glucose	200 mg	2.0 g
100 mg/mL Ampicillin	0.05 mL	0.5 mL

7. 1X M9 Minimal Salts ($^2H^{13}C^{15}N$-Labelled)

Reagents	1000 mL
Sodium Phosphate Dibasic (Na_2HPO_4)	6.8 g
Potassium Phosphate Monobasic (KH_2PO_4)	3.0 g
Sodium Chloride (NaCl)	0.5 g
Deuterium Oxide	1000 mL

a) Do not adjust the pH.
b) Autoclave.

8. 1X M9 Minimal Medium ($^2H^{13}C^{15}N$-LABELLED)

Reagent	100 mL	1000 mL
1X M9 Minimal Salts ($^2H^{13}C^{15}N$-LABELLED)	100 mL	900 mL
0.1 M Calcium Chloride ($CaCl_2$)	0.1 mL	1.0 mL
1 M Magnesium Sulfate ($MgSO_4$)	0.1 mL	1.0 mL
MEM Vitamins Solution	1.0 mL	10.0 mL
^{15}N Ammonium Chloride ($^{15}NH_4Cl$) or ^{15}N Ammonium Sulfate ($^{15}NH_4)_2SO_4$	100 mg	1.0 g
Glucose $U^{13}C_6$ or $U^{13}C_6$-1,2,3,4,5,6,6-D_7	200 mg	2.0 g
100 mg/mL Ampicillin	0.05 mL	0.5 mL

B. Methods

This protocol is for a 1-liter culture.

---

1. Preparation

a) Prepare all solutions.

b) Autoclave as follows:

(1) *Medium Luria-Bertani (LB)*

(2) *Agar-LB Plates*

(3) *1 M Magnesium Sulfate ($MgSO_4$)*

(4) *0.1 M Calcium Chloride ($CaCl_2$)*

(5) *One 2.8-liter Erlenmeyer baffled cell culture flask with 800 mL of milliQ-water*

(6) *One 2.8-liter Erlenmeyer baffled cell culture flask with 1000 mL of 1X M9 Minimal Salts ($^2H^{13}C^{15}N$-LABELLED)*

(7) *10X M9 Minimal Salts (Natural Abundance)*

(8) *One empty 1000-mL Erlenmeyer baffled cell culture flask*

(9) *One 1000-mL Erlenmeyer baffled cell culture flask with 90 mL of milliQ-water*

2. Transformation

a) For each protein (light and heavy chain), freshly transform ~50 ng of DNA into 50-100 µL of **competent *Escherichia coli* BL21(DE3) cells**.

(1) *Incubate on ice for 30 minutes*

(2) *Heat shock at 42°C for 2 minutes*

(3) *Leave on ice for 5 minutes*

(4) *Add 1 mL of **Medium Luria-Bertani (LB)** and incubate for 30-60 minutes at 37°C with agitation*

(5) *Spin down cells for 1 minute at 1.5 rcf*

(6) *Remove the excess liquid - keep ~200 µL*

(7) *Spread bacteria on an* ***Agar-LB plate***

b) Incubate overnight (reverse) at 37°C.

3. Initiating the Pre-Culture

a) In the morning, pick one colony of the Light Chain (LC) and inoculate 4-5 mL of Medium Luria-Bertani (LB) (containing the antibiotic).

b) Incubate the colony for 5-7 hours (or until the end of the day) at 37°C (225 rpm).

c) Repeat steps a) and b) for the Heavy Chain Fab (HC-Fab).

d) For the natural abundance chain, prepare 100 mL of **1X M9 Minimal Medium (Natural Abundance)** into the "1000-mL Erlenmeyer baffled cell culture flask with 90 mL of milliQ-water" previously prepared in section I.B.1.

e) For the labelled chain, transfer 100 mL of **1X M9 Minimal Salts ($^2H^{13}C^{15}N$-LABELLED)** into the "empty 1000-mL Erlenmeyer baffled cell culture flask" previously prepared in section I.B.1. Then, prepare 100 mL of ***1X M9 Minimal Medium ($^2H^{13}C^{15}N$-LABELLED)***.

f) Add 100 µL of the previously inoculated LB culture (natural abundance chain) into the freshly prepared **1X M9 Minimal Medium (Natural Abundance)**.

g) Add 100 µL of the previously inoculated LB culture (labelled chain) into the freshly prepared ***1X M9 Minimal Medium ($^2H^{13}C^{15}N$-LABELLED)***.

h) Incubate overnight at 37°C (225 rpm).

4. Protein Expression (Next Day)

a) Prepare 900 mL of **1X M9 Minimal Medium (Natural Abundance)** into the "2.8-liter Erlenmeyer baffled cell culture flask with 800 mL of milliQ-water" previously prepared in section I.B.1.

b) Take the optical density at 600 nm of the overnight pre-cultures.

c) Transfer the entire content (100 mL) of the overnight **1X M9 Minimal Medium (Natural Abundance)** pre-culture into the freshly prepared 900 mL of **1X M9 Minimal Medium (Natural Abundance)**.

d) Prepare 900 mL of ***1X M9 Minimal Medium ($^2H^{13}C^{15}N$-LABELLED)*** into the "2.8-liter Erlenmeyer baffled cell culture flask with 1000 mL of 1X M9 Minimal Salts ($^2H^{13}C^{15}N$-LABELLED)" previously prepared in section I.B.1. You should have 900 mL left after taking out 100 mL in section I.B.3.

e) Take the optical density at 600 nm of the overnight pre-cultures.

f) Transfer the entire content (100 mL) of the overnight **1X M9 Minimal Medium ($^2H^{13}C^{15}N$-LABELLED)** pre-culture into the freshly prepared 900 mL of **1X M9 Minimal Medium ($^2H^{13}C^{15}N$-LABELLED)**.

g) Incubate at 37°C (225 rpm).

h) To assign the Isoleucine, Leucine, and Valine (ILV), monitor the optical density at 600 nm in the first 2-3 hours of the culture.

i) When the optical density at 600 nm reaches 0.4, add to the **1X M9 Minimal Medium ($^2H^{13}C^{15}N$-LABELLED)**:

(1) 25 mg of α-**Ketobutyric Acid-^{13}C-3,3-D_2** (Cambridge Isotope Laboratories, CDLM-4611)

(2) 50 mg of **α-Ketoisovaleric-U-$^{13}C_5$ Acid-3-D_1** (Cambridge Isotope Laboratories, CDLM-4418)

j) Incubate at 37°C (225 rpm).

k) When the optical density at 600 nm reaches 0.8, add 1 mM (final concentration) of **Isopropyl-beta-D-thiogalactopyranoside (IPTG)**. Record the optical density at 600 nm.

The culture in natural abundance should grow faster. As a consequence, they won't reach 0.8 at the same time.

---

l) For each flask, transfer 1.0 mL of culture (t=0) to a microtube. Spin down the cells for 1 minute at 9.3 rcf. Immediately resuspend the pellet in 200 µL of **2X Gel Loading Buffer**. Keep at -20°C for later (for the SDS-PAGE gel).

m) Incubate the cultures for 24 hours.

n) Once completed, record the optical density at 600 nm.

o) For each flask, transfer 1.0 mL of culture (t=24) to a microtube. Spin down the ells for 1 minute at 9.3 rcf. Immediately resuspend the pellet in 200 µL of **2X Gel Loading Buffer**. Keep at -20°C for later (for the SDS-PAGE gel).

p) Transfer the content of each flask into a large (1 L) centrifugation bottle.

q) Spin down the cells 30 minutes (4°C) at 3,000 rcf.

r) Transfer the supernatant of the **1X M9 Minimal Medium (Natural Abundance)** culture back into its 2.8-liter Erlenmeyer baffled cell culture flask.

s) Resuspend the pellet with 20 mL of supernatant. Transfer to a 50-mL conical tube.

t) Add another 20 mL of supernatant to the pellet and collect any remaining cells. Add it to the 50-mL conical tube.

u) Repeat steps r), s), and t) for the **1X M9 Minimal Medium ($^2H^{13}C^{15}N$-LABELLED)** culture.

v) Centrifuge the resuspended pellets for 60 minutes at 3,220 rcf.

w) Discard the supernatant.

x) Weigh the pellet and store at -80°C.

y) Check protein expression on SDS-PAGE gel:

(1) Recommended gel for a 25-75 kDa protein: **Stain Free AnyKD (Bio-Rad)**

(2) Take out the aliquots stored at -20°C

(3) Heat samples at 99°C for 10 minutes, mix by inversion

(4) Load 5 µL (when still warm) per lane on the gel (if you are using a 15-µL gel comb)

(5) Run the gel at 200 volts for 30 minutes, or as needed

(6) Visualize the proteins on the gel

II. PROTEIN REFOLDING AND PURIFICATION

A. PREPARATION OF SOLUTIONS

1. BUFFER LYSIS

**Reagents**	**1000 mL**
Tris Base (MW 121.14)	1.68 g
Tris Hydrochloride (MW 157.60)	0.97 g
500 mM Ethylenediaminetetraacetic Acid (EDTA) at pH 8.0 (2 mM final)	4 mL
Dithiothreitol (DTT) (10 mM final)	1.54 g
milliQ-water	950 mL

a) Adjust the pH to 8.5 with Hydrochloric Acid (HCl) or Sodium Hydroxide (NaOH).
b) Complete the volume to 1000 mL with milliQ-water.
c) Filter on a 0.22 µm membrane.

2. BUFFER RB (REFOLDING BUFFER)

We recommend using a 1 M L-Arginine Monohydrochloride concentration. Lower yields were obtained with 0.5 M L-Arginine Monohydrochloride, while 2 M did not increase the yield significantly.

**Reagents**	**1000 mL**
Tris Base (100 mM final)	12.1 g
500 mM Ethylenediaminetetraacetic Acid (EDTA) at pH 8.0 (2 mM final)	4 mL
L-Arginine Monohydrochloride (1 M)	210.66 g
milliQ-water	500 mL

a) Complete the volume to 800 mL with milliQ-water.
b) Cool down the buffer at 4°C for at least 3 hours, ideally overnight.
c) An hour prior injection, add the Glutathione Oxidized.

**Reagents**	**1000 mL**
L(-)-Glutathione Oxidized (3.2 mM final)	1.96 g

d) Adjust the pH to 10.0 with 10 N Sodium Hydroxide (NaOH). Note that you might need 50-100 mL of NaOH to reach to desired pH.

Alternatively, NaOH pellets can be used. However, because it takes time to dissolve, it is less practical than using NaOH in solution.

---

e) Complete the volume to 1000 mL with milliQ-water.

A pH of 9-10 is recommended. Reduced yields were obtained with a pH below 9.

---

3. Buffer PSB (Protein Solubilization Buffer)

**Reagents**	**5 mL**
Tris Base (100 mM final)	0.24 g
milliQ-water	5 mL

a) Decrease the pH with 4-5 drops of Glacial Acetic Acid.

**Reagents**	**20 mL**
500 mM Ethylenediaminetetraacetic Acid (EDTA) at pH 8.0 (2 mM final)	80 µL
Guanidium Hydrochloride (6 M final)	11.5 g
Glutathione Reduced (80 mM)	0.49 g

b) Complete the volume to 20 mL with milliQ-water.
c) Adjust the pH to 8.5 with solid Tris Base.

4. Buffer CS (20 mM Tris)

**Reagents**	**1000 mL**
Tris-Base	271.6 mg
Tris-HCl	2.8 g
milliQ-water	950 mL

a) Adjust the pH to 7.0-7.5 with Hydrochloric Acid (HCl) or Sodium Hydroxide (NaOH).
b) Complete the volume to 1000 mL with milliQ-water.
c) Filter on a 0.22 µm membrane.

5. Buffer Elution #1 (Acetic Acid)

Reagents	1000 mL
17.4 M Acetic Acid (0.5 M Final)	28.7 mL
milliQ-water	971.3 mL

a) Filter on a 0.22 µm membrane.

6. Buffer Elution #2 (Glycine-HCl)

Reagents	1000 mL
Glycine (0.1 M final)	7.5 g
milliQ-water	950 mL

a) Adjust the pH to 2.5 with Hydrochloric Acid (HCl).
b) Complete the volume to 1000 mL with milliQ-water.
c) Filter on a 0.22 µm membrane.

7. Buffer SP

Reagents	1000 mL
Sodium Acetate (20 mM Final)	1.74 g
milliQ-water	950 mL

a) Adjust the pH to 5.0 with Glacial Acetic Acid.
b) Complete the volume to 1000 mL with milliQ-water.
c) Filter on a 0.22 µm membrane.

8. Buffer SPE

Reagents	1000 mL
Sodium Acetate (20 mM Final)	1.74 g
Sodium Chloride (NaCl) (1 M Final)	58.4 g
milliQ-water	950 mL

a) Adjust the pH to 5.0 with Glacial Acetic Acid.
b) Complete the volume to 1000 mL with milliQ-water.
c) Filter on a 0.22 µm membrane.

9. Buffer NMR

Reagents	1000 mL
Sodium Acetate-d_3 (20 mM Final)	1.74 g
milliQ-water	950 mL

a) Adjust the pH to 5.0 with Glacial Acetic Acid.
b) Complete the volume to 1000 mL with milliQ-water.
c) Filter on a 0.22 µm membrane.

B. METHODS

Buffer RB's ratio protein/volume should be 50-100 µg/mL. Assuming there is roughly 100 mg of protein per liter of culture (approximately 2.5 g cell pellet), we recommend using 2 L of Buffer RB for a 1-L culture. Therefore, 20 mL of Buffer PSB will be used, giving a 4:1 ratio of Oxidized/Reduced Glutathione during the refolding.

---

1. CELL LYSIS & PROTEIN SOLUBILIZATION

a) The previous day, prepare **Buffer RB** without the **Oxidized Glutathione** - At this point, do not adjust the pH. Store at 4°C overnight under agitation.
   (1) *The volume of Buffer RB to prepare will depend on the weight of the cell pellet being used. Check the note above.*
b) Take out a frozen cell pellet for LABELLED HC-Fab (or LABELLED LC) and UNLABELLED LC (or UNLABELLED HC-Fab) of expressed cultures:
   (1) *The cell pellet weight to use will vary for each protein. Generally, a cell pellet prepared from 0.5-1.0 liters of culture should be sufficient.*
   (2) *The **Light Chain (LC)** should be in excess (1.25-2.0).*
c) Fill the 50-mL conical tube (containing the pellet) with **Buffer Lysis** up to the 35 mL mark.
d) Sonicate (microtip probe) each pellet in an ice/cold water container at 40% intensity: 5 sec ON, 5 sec OFF, 5 minutes.
   (1) *When the sonication is completed, cool down the sample for 10 minutes.*
   (2) *Repeat the sonication 2 more times.*

The sonication conditions should be adjusted if a different probe is used. Alternatively, a French Press can be used to lyse cells.

---

e) Take an aliquot of each cell lysate. Mix 10 µL of the sample with 90 µL of **2x Gel Loading buffer**.
f) Centrifuge cell lysates for 30 minutes (4°C) at 30,310 rcf.
g) During the centrifugation, prepare the **Buffer PSB**.

h) Transfer each supernatant to clean tubes.

i) Take an aliquot of each supernatant. Mix 10 µL of the sample and add 90 µL of **2x Gel Loading buffer**.

j) Resuspend each pellet separately in 10 mL of **Buffer PSB**. Use a small beaker (e.g., 50 mL) to resuspend the pellet.

  (1) *Add the buffer to the pellet and scrape it with a spatula before transferring the contents into the beaker.*

k) Resuspend each pellet for 2-3 hours (or until the pellet is completely resuspended) at 4°C under agitation.

l) Take an aliquot of each pellet. Mix 10 µL of the sample and add 90 µL of **2x Gel Loading buffer**.

m) Check protein content on SDS-PAGE gel:

  (1) *Recommended gel for a 25-75 kDa protein:* ***Stain Free AnyKD (Bio-Rad)***

  (2) *Heat gel samples at 99°C for 10 minutes*

  (3) *Load 5 µL (when still warm) of each sample on a gel*

    (a) *Due to the presence of a high concentration of Glutathione Hydrochloride, samples should be loaded when still "warm", which is particularly important for the pellet aliquot*

  (4) *Run the gel at 200 volts for 30 minutes, or as needed*

  (5) *Visualize the proteins on the gel*

n) In parallel, prepare a 1:100 dilution (protein:milliQ-Water) of each pellet to estimate the concentration using the optical density at 280 nm. Blank with milliQ-Water.

o) Based on the estimated concentration and band intensity on the gel, the ratio of HC-Fab **to** LC can be calculated.

2. Protein Refolding and Purification

a) Mix the resuspended pellets to the desired ratio.

b) Add the **Oxidized Glutathione** to the previously prepared **Buffer RB** and adjust the pH to 10.0 (4°C) with **10 N Sodium Hydroxide (NaOH)**.

c) Use a syringe pump to slowly inject the resuspended mixture into the **RB Buffer** at 4°C, stirring continuously.

  (1) *Aspirate the sample with a syringe*

  (2) *Set it on the pump*

  (3) *Set the pump with the correct sample volume, plus a few more milliliters*

  (4) *Set the rate at 30 mL/hr*

  (5) *Run the pump until the sample is injected*

Alternatively, the mixture can be slowly added drop-by-drop to Buffer RB.

---

d) Leave the solution at 4°C for 48-72 hours under slow agitation.

Incubation up to 5 days worked as well - we did not test it above this period. However, it is recommended to refold for at least 48 hours.

---

e) Dialyze each liter against 4 L of **100 mM Tris Hydrochloride** (do not pH), with 5 buffers exchanged.
   (1) *It is recommended to use* ***Snakeskin Dialysis Tubing, 35mm, 10 MWCO (Thermo Fisher Scientific)*** *because they are ready-to-use, and they do not need to be pre-wet, as most dialysis bags do*
   (2) *Buffer exchanges are usually done as follows: Day 1 at 9 am, 12 pm, and 5 pm; Day 2 at 9 am and 12 pm*
   (3) *The target pH is 7.0-7.5 for the CaptureSelect™ CH1-XL Affinity Matrix (Thermo Fisher Scientific)*
   (4) *The target pH is usually reached after 5 buffer exchanges*
   (5) *Open one bag (take the smallest one) and check the pH - If the pH hasn't been reached, continue with more dialysis*

f) Once completed, remove the protein from dialysis bags.

g) Centrifuge for 5 minutes at 26,640 rcf to remove aggregates. Decant the supernatant.

h) Add a filtering step using a 5-10 µm membrane if the solution remains unclear.

i) Add a filter on the injection line to prevent aggregates from entering the FLPC Chromatography System for extra safety.

j) Prepare a **CaptureSelect™ CH1-XL Affinity Matrix** (Thermo Fisher Scientific) column (10 mL of resin is recommended).

k) Inject the protein into the **CaptureSelect™ CH1-XL Affinity Matrix** column (using an FPLC):
   (1) *Equilibration in* ***Buffer CS*** *(5 CVs, 4 mL/min)*
   (2) *Injection (Injection pump, 4 mL/min)*
   (3) *Wash with* ***Buffer CS*** *(5 CVs, 4 mL/min)*
   (4) *Elution with* ***Elution Buffer #1*** *(10 CVs, 4 mL/min)*
   (5) *Elution with* ***Elution Buffer #2*** *(10 CVs, 4 mL/min)*
   (6) *Clean-up with* ***Buffer CS*** *(5 CVs, 4 mL/min)*

l) Pool fractions containing the protein.

m) Dialyzed 2-3 times (3-4 hours each) against **Buffer SP**. Check the pH to confirm that the buffer exchange worked - pH should be approximately 5.0.

n) Prepare an **SP Sepharose™ Fast Flow Cation Exchange** (Cytiva) column (10 mL of resin is recommended).

o) Inject the protein into an **SP Sepharose™ Fast Flow Cation Exchange** column (using an FPLC):

(1) *Equilibration in **Buffer SP** (5 CVs, 4 mL/min)*
(2) *Injection (Injection pump, 4 mL/min)*
(3) *Wash with **Buffer SP** (5 CVs, 4 mL/min)*
(4) *Elution with **Buffer SPE** (10 CVs gradient, 4 mL/min)*
(5) *Clean-up with **Buffer SP** (5 CVs, 4 mL/min)*

p) Pool fractions containing the protein.

q) Concentrate the protein on **Amicon Ultra-15 (10 kDa)** and buffer exchanged in **Buffer NMR**.

r) Estimate protein concentration by NanoDrop.

(1) *Protein concentration targeted: <u>250-750 μM</u>*

3. PREPARE PROTEINS FOR NMR

a) Add 5% **Deuterium Oxide (D_2O)** to proteins for NMR.

b) Add 2-5 mM (recommended) of Sodium 2,2-dimethyl-2-silapentane-5-sulfonate (DSS) for referencing.

c) Transfer to an **NMR tube**.

Supplemental Figure 1: 2D-^{1}H-^{15}N-HSQC NMR spectra of ^{15}N-adalimumab-Fab, ^{15}N-bevacizumab-Fab, ^{15}N-infliximab-Fab, ^{15}N-rituximab-Fab, and ^{15}N-trastuzumab-Fab, all in 20 mM NaOAc-d_3 pH 5.0 recorded at 700 MHz at 40 ^{o}C.

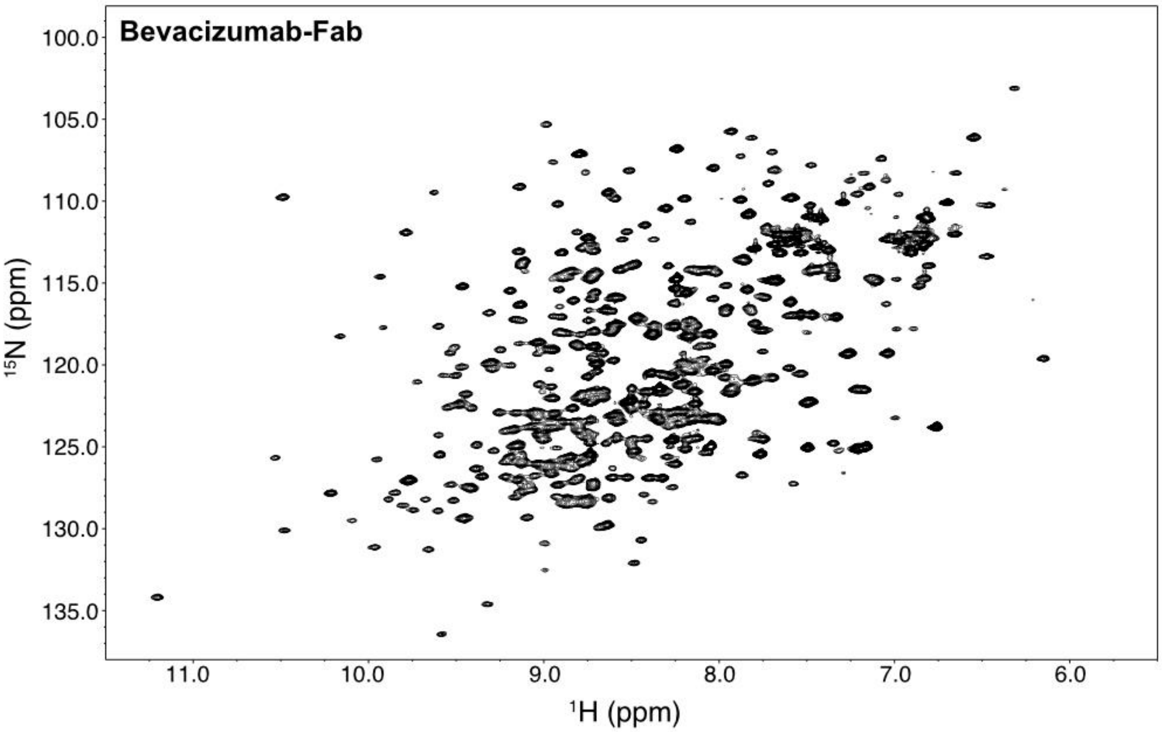

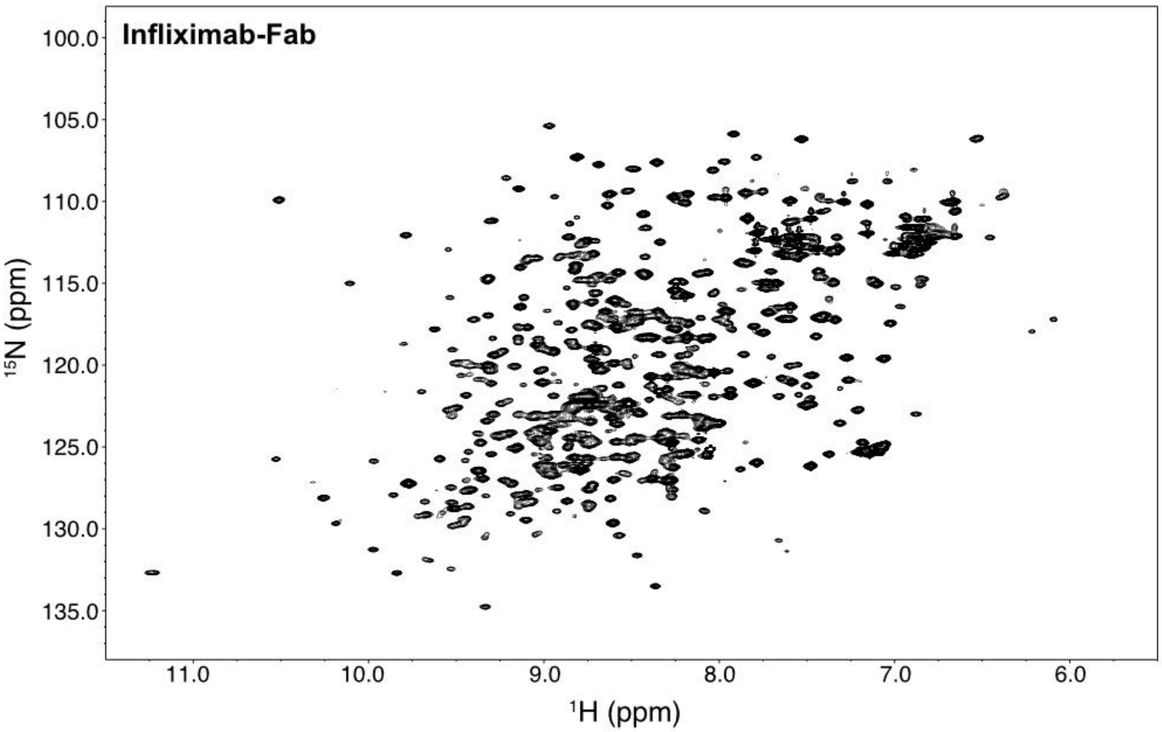

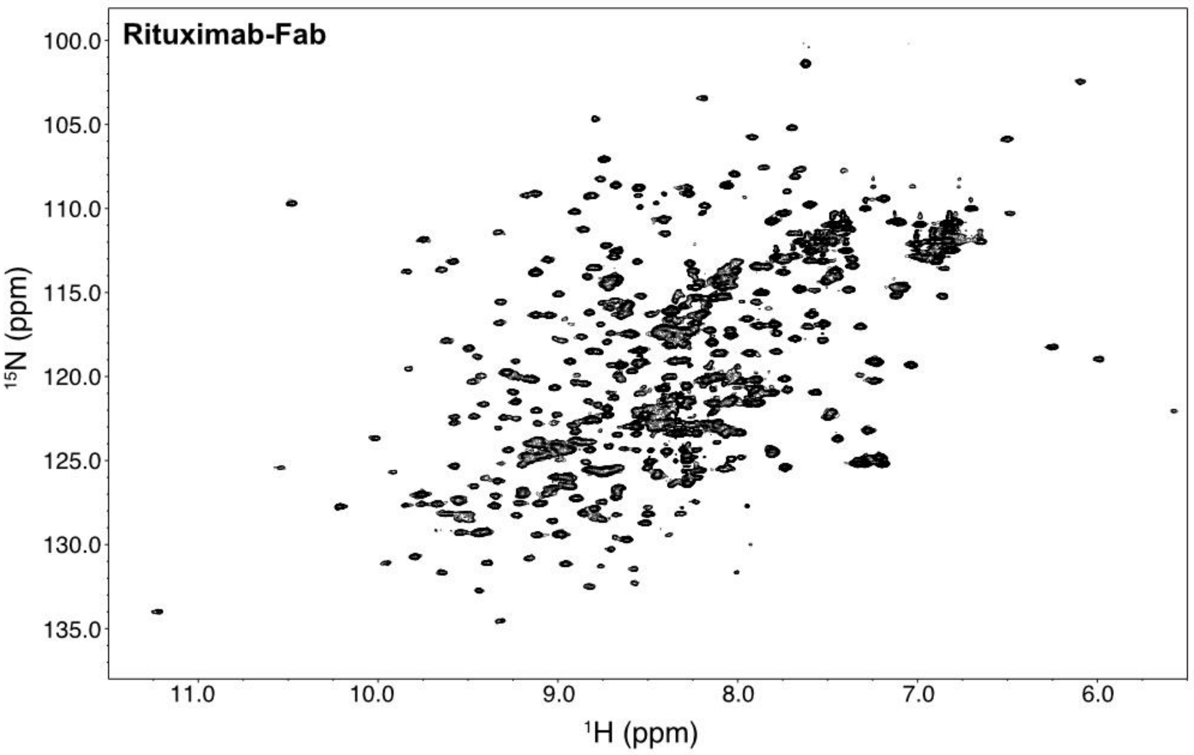
Rituximab-Fab
100.0
105.0
110.0
115.0
120.0
125.0
130.0
135.0
^{15}N (ppm)
11.0
10.0
9.0
8.0
7.0
6.0
^{1}H (ppm)

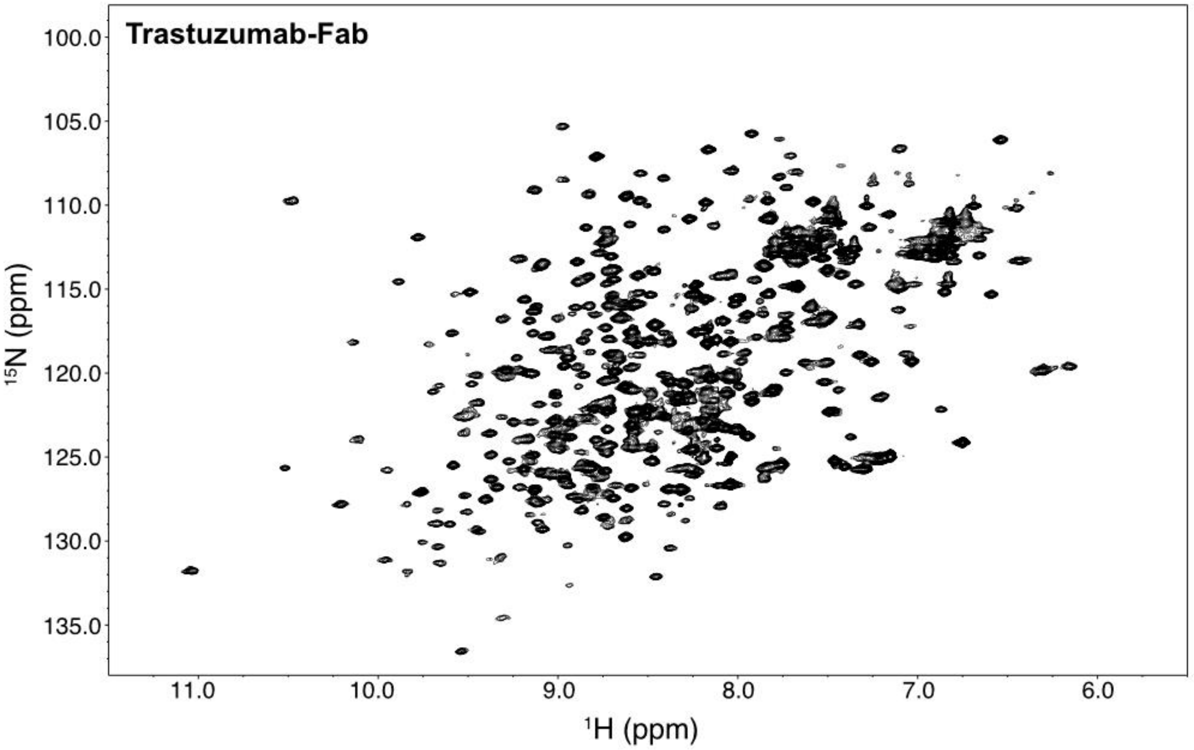
Trastuzumab-Fab
100.0
105.0
110.0
115.0
120.0
125.0
130.0
135.0
^{15}N (ppm)
11.0
10.0
9.0
8.0
7.0
6.0
^{1}H (ppm)

Supplemental Figure 2: 2D-^{1}H-^{15}N-HSQC NMR spectra of ^{15}N-adalimumab-Fab, in 20 mM NaOAc-d_3 pH 5.0 recorded at 700 MHz at 40 °C. Top panel is the overlay of the spectra of the ^{15}N-labelled on the heavy in black and the ^{15}N-labelled on the light chain in red. The middle and bottom panel show the spectra of the heavy and light chains, respectively.

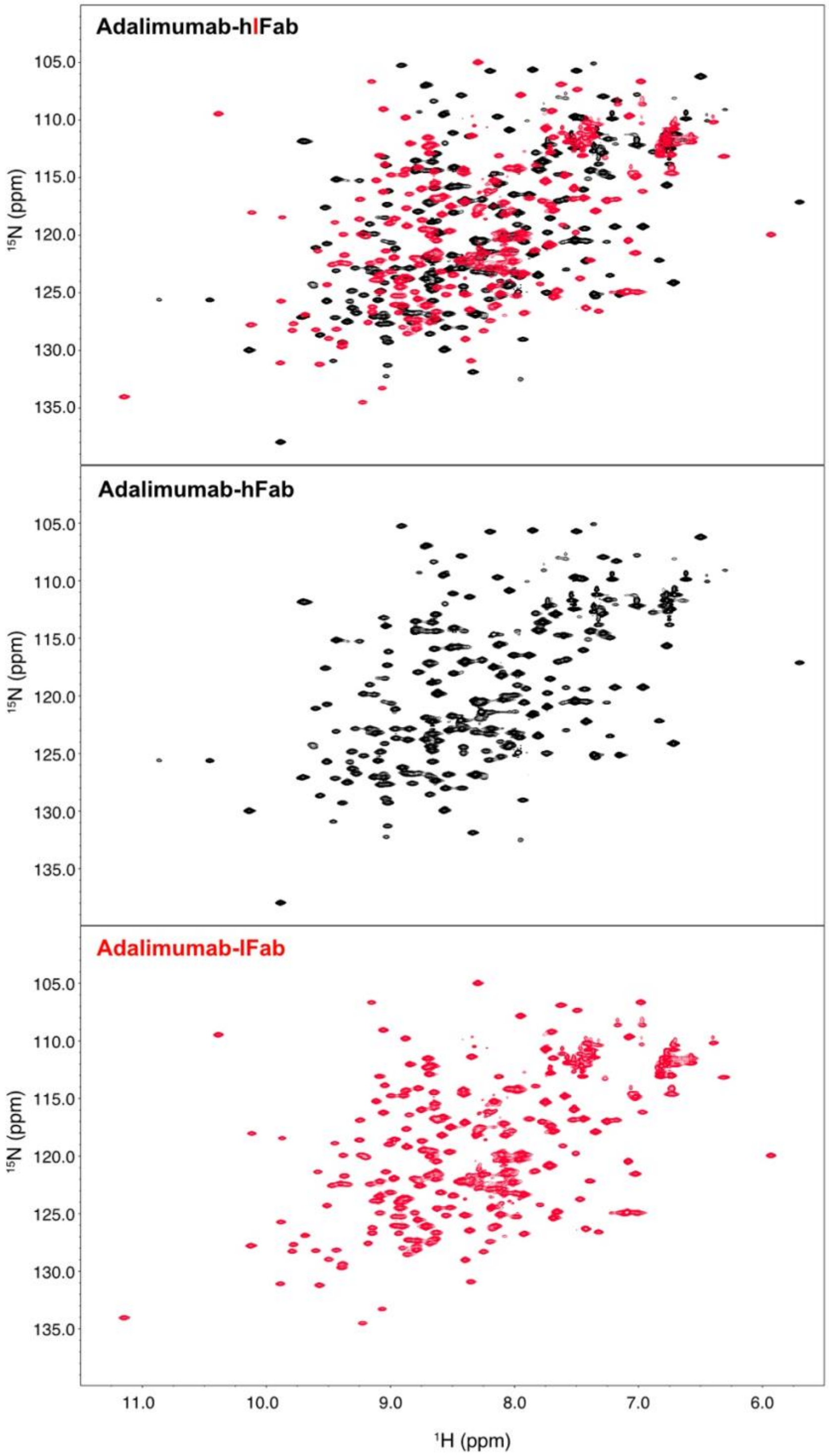

Supplement: Supplementary file 1 [file ac6c00109_si_001.pdf]
